# Carbapenem-resistant hypervirulent ST23 *Klebsiella pneumoniae* with a highly transmissible dual-carbapenemase plasmid in Chile

**DOI:** 10.1186/s40659-024-00485-2

**Published:** 2024-03-12

**Authors:** Matías Gálvez-Silva, Patricio Arros, Camilo Berríos-Pastén, Aura Villamil, Paula I. Rodas, Ingrid Araya, Rodrigo Iglesias, Pamela Araya, Juan C. Hormazábal, Constanza Bohle, Yahua Chen, Yunn-Hwen Gan, Francisco P. Chávez, Rosalba Lagos, Andrés E. Marcoleta

**Affiliations:** 1 Grupo de Microbiología Integrativa, Laboratorio de Biología Estructural y Molecular BEM, Departamento de Biología, Facultad de Ciencias, Universidad de Chile Las Palmeras, Ñuñoa, Santiago, 3425 Chile; 2 Laboratorio de Microbiología de Sistemas, Departamento de Biología, Facultad de Ciencias, Universidad de Chile Las Palmeras, Ñuñoa, Santiago, 3425 Chile; 3 Instituto de Salud Pública Marathon, Ñuñoa, Santiago, 1000 Chile; 4 Yong Loo Lin School of Medicine, National University of Singapore, MD7, 8 Medical Drive, Singapore, Singapore; 5 Hospital San José, Santiago, Chile

**Keywords:** *Klebsiella pneumoniae*, Hypervirulence, Carbapenem resistance, Convergence, Conjugative plasmids, Mobile genetic elements

## Abstract

**Background:**

The convergence of hypervirulence and carbapenem resistance in the bacterial pathogen *Klebsiella pneumoniae* represents a critical global health concern. Hypervirulent *K. pneumoniae* (hvKp) strains, frequently from sequence type 23 (ST23) and having a K1 capsule, have been associated with severe community-acquired invasive infections. Although hvKp were initially restricted to Southeast Asia and primarily antibiotic-sensitive, carbapenem-resistant hvKp infections are reported worldwide. Here, within the carbapenemase production Enterobacterales surveillance system headed by the Chilean Public Health Institute, we describe the isolation in Chile of a high-risk ST23 dual-carbapenemase-producing hvKp strain, which carbapenemase genes are encoded in a single conjugative plasmid.

**Results:**

Phenotypic and molecular tests of this strain revealed an extensive resistance to at least 15 antibiotic classes and the production of KPC-2 and VIM-1 carbapenemases. Unexpectedly, this isolate lacked hypermucoviscosity, challenging this commonly used hvKp identification criteria. Complete genome sequencing and analysis confirmed the K1 capsular type, the KpVP-1 virulence plasmid, and the GIE492 and ICEKp10 genomic islands carrying virulence factors strongly associated with hvKp. Although this isolate belonged to the globally disseminated hvKp clonal group CG23-I, it is unique, as it formed a clade apart from a previously reported Chilean ST23 hvKp isolate and acquired an IncN KPC-2 plasmid highly disseminated in South America (absent in other hvKp genomes), but now including a class-I integron carrying *bla*_VIM−1_ and other resistance genes. Notably, this isolate was able to conjugate the double carbapenemase plasmid to an *E. coli* recipient, conferring resistance to 1st -5th generation cephalosporins (including combinations with beta-lactamase inhibitors), penicillins, monobactams, and carbapenems.

**Conclusions:**

We reported the isolation in Chile of high-risk carbapenem-resistant hvKp carrying a highly transmissible conjugative plasmid encoding KPC-2 and VIM-1 carbapenemases, conferring resistance to most beta-lactams. Furthermore, the lack of hypermucoviscosity argues against this trait as a reliable hvKp marker. These findings highlight the rapid evolution towards multi-drug resistance of hvKp in Chile and globally, as well as the importance of conjugative plasmids and other mobile genetic elements in this convergence. In this regard, genomic approaches provide valuable support to monitor and obtain essential information on these priority pathogens and mobile elements.

**Supplementary Information:**

The online version contains supplementary material available at 10.1186/s40659-024-00485-2.

## Background

The rise of bacterial pathogens displaying multi-drug resistance and increased virulence is currently one of the most pressing threats to global health. Among them is the Gram-negative rod-shaped bacteria *Klebsiella pneumoniae* (*Enterobacterales*), one of the most prevalent agents causing multi-drug resistant infections worldwide and a major trafficker of antibiotic resistance genes [1, 2]. In particular, hypervirulent *K. pneumoniae* (hvKP) causing severe community-acquired metastatic infections in healthy individuals, and especially hvKp strains resistant to last-resort carbapenem antibiotics (CR-hvKp), are a critical concern [3, 4]. Although the number of cases is still relatively limited, these convergent CR-hvKp strains have been shown to arise through different evolutionary pathways, including hvKp acquiring carbapenemase plasmids, CR-Kp acquiring virulence determinants, or by the acquisition of hybrid plasmids encoding carbapenemases and hypervirulence determinants [5, 6].

Phylogenomic analyses showed that most hvKp strains are from sequence type (ST) and clonal group (CG) 23, mainly from the globally disseminated CG23-I subgroup. Also, they tend to have K1 or K2 capsular serotypes and an expanded array of virulence factors encoded in mobile genetic elements [7–9]. Among them, the large virulence plasmid (KpVP) carries genes for the synthesis, secretion, and uptake of aerobactin and salmochelin, two siderophores for iron scavenging, the *rmpACD* genes linked to the hypermucoviscous capsule, and genes involved in metal resistance [10]. Additionally, they harbor the integrative-conjugative element ICE*Kp*10, including genes for producing yersiniabactin siderophores and the genotoxin colibactin. Also, most hvKp have the GIE492 genomic island carrying the genes for producing the antibacterial peptide microcin E492 and salmochelin [11, 12].

While ST23 CR-hvKP strains from several countries, including China [13], Singapore [14], Germany [15], and the USA [16], have been reported and characterized, there are limited reports from other regions, such as Latin America [17, 18], especially at a genomic level. Therefore, monitoring the emergence and spread of these strains is essential to develop public health strategies and guide interventions for their prevention and control. Towards this direction, we report the isolation of an ST23 dual-carbapenemase-producing hvKp strain from a respiratory tract infection in Chile. We combined genomics analyses and phenotypic tests to obtain relevant information on this high-risk isolate.

## Results and discussion

### Phenotypic antibiotic resistance profile of *K. pneumoniae* VA585-22

The VA585-22 strain was isolated on September 29, 2022, in a hospital in Santiago, Chile, from the tracheal aspirate of a 31-year-old gun-injured male after a prolonged hospitalization in the intensive care unit with pneumonia and mechanical ventilation. Antimicrobial susceptibility testing showed resistance to 3rd, 4th, and 5th -generation cephalosporins, aminopenicillins, carbapenems, and aminoglycosides (Table 1). Also, this isolate showed intermediate resistance to colistin and levofloxacin. The presence of *bla*_KPC−2_ and *bla*_VIM−1_ genes, encoding the KPC-2 serine carbapenemase and the VIM-1 metallo-carbapenemase, was detected by PCR. Furthermore, the expression of the carbapenemases was confirmed by immunochromatography (Table 1).

**Table 1:** Antimicrobial susceptibility profile and detection of carbapenemase genes and its expression in *K. pneumoniae* VA585-22 and *E. coli* K12 transconjugants that acquired pVA585-22_54

	VA585-22	K12(Gen^R^)	K12-1	K12-2
Antibiotic class Antibiotic	MIC (µg/mL)	MIC (µg/mL)	MIC (µg/mL)	MIC (µg/mL)
AminopenicillinAmpicillin	> 16R	8S	> 16R	> 16R
Ampicillin/Sulbactam	> 16/8R	≤ 4/2S	> 16/8R	> 16/8R
Piperacillin/Tazobactam	> 32/4R	≤ 4/4S	> 64/4R	> 64/4R
Cephalosporin 1st Cephazolin	> 16R	≤ 2S	> 16R	> 16R
Cephalosporin 2nd Cefoxitin Ceftriaxone Cephalosporin 3rd Ceftazidime	> 16R > 4R > 16R	≤ 4S ≤ 0.5S ≤ 0.5S	> 16R > 4R > 16R	> 16R > 4R > 16R
Ceftazidime/Avibactam Cephalosporin 4th Cefepime	> 16/4R > 16R	≤ 0.25/4S 2S	> 16/4R > 16R	> 16/4R > 16R
Cephalosporin 5th Ceftolozane/Tazobactam	> 32/4R	≤ 1/4S	> 32/4R	> 32/4R
MonobactamAztreonam Ertapenem CarbapenemMeropenem	16R > 2R 8R	≤ 1S ≤ 0.25S ≤ 0.5S	16R 2R 2I	16R 2R 4R
Imipenem	16R	1S	8R	8R
TetracyclineMinocycline	2S	4S	2S	2S
GlicilcyclineTigecycline	≤ 0.25NI	≤ 1NI	≤ 1NI	≤ 1NI
AminoglycosideAmikacin	16R	32R	> 32R	> 32R
Gentamicin	≤ 0.5S	8R	> 8R	> 8R
Fluoroquinolone 2nd Ciprofloxacin	≤ 0.064S	≤ 0.125S	≤ 0.125S	≤ 0.125S
Norfloxacin Fluoroquinolone 3rd Levofloxacin	≤ 2S ≤ 1I	≤ 2S ≤ 1I	≤ 2S ≤ 1I	≤ 2S ≤ 1I
Other Fosfomycin Colistin	≤ 16S 0.5I	≤ 16S ≤ 1I	≤ 16S 0.5I	≤ 16S 0.5I
^**1**^ **Carbapenemase gene** *bla*_KPC_	*+*	*-*	*+*	*+*
*bla*VIM	*+*	*-*	*+*	*+*
^**2**^ **Carbapenemase expression**				
VIM-1	+	-	+	+
KPC-2	+	-	+	+

^1^The carbapenemase genes were detected by PCR using specific primers. (+): present; (-): absent

^2^The carbapenemase expression was assessed by immunochromatography. (+): detected; (-): not detected

### Genomic virulence, antibiotic resistance, and mobile genetic elements

**Fig. 1:**
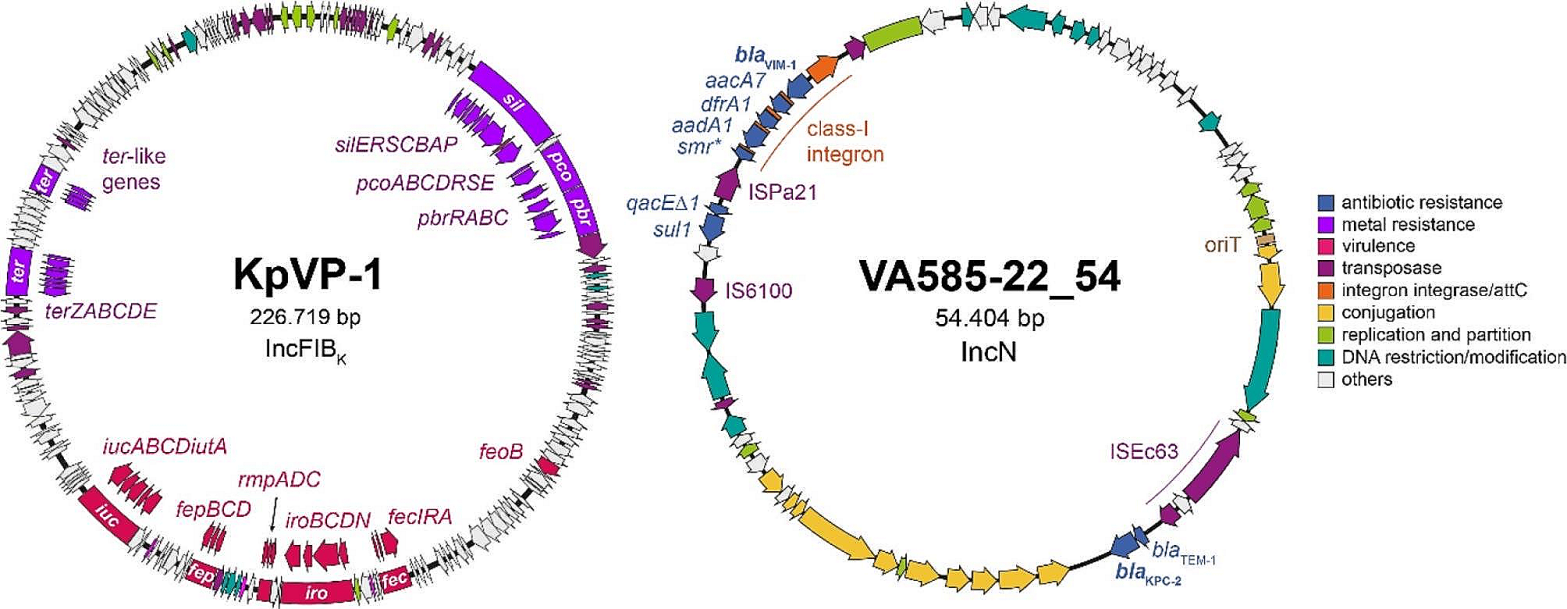
Main features of the plasmids present in the hypervirulent ST23 *K. pneumoniae* strain VA585-22. The carbapenemase genes are shown in bold

The VA585-22 complete genome was sequenced and assembled (Table S1), which consisted of three circular replicons, a 5,334,759 bp chromosome, and two plasmids. One corresponded to a KpVP-1-like large virulence plasmid (~ 227 kbp, IncFIB_K_) encoding several iron acquisition systems, including the siderophore gene clusters *iucABCDiutA* (aerobactin) and *iroBCDN* (salmochelin), the *fepBCD* ABC-type iron transporter, and the Fur-dependent regulatory system for iron uptake *fecIRA* (Fig. 1, left side). Also, this plasmid included the *sil*, *pco*, and *ter* genes linked with resistance to copper, silver, lead, and tellurite [10, 19] and the *rmpADC* genes linked to hypermucoviscosity [20]. Unexpectedly, VA585-22 lacked hypermucoviscosity, as revealed by low-speed sedimentation assays and the string test (Fig. S1). We used Kp SGH10, a hypermucoviscous strain proposed as an hvKp representative [7], and the D*wcaJ* capsule-null mutant lacking hypermucoviscosity [21] as controls. A closer examination of the *rmpADC* locus indicated 100% amino acid identity of RmpA and RmpC, comparing VA585-22 with SGH10. Moreover, the gene organization of this operon and its immediate surroundings are conserved between both strains, including a 100% nucleotide identity in the promoter region (Fig. S2). Conversely, RmpD had a D3E substitution, which, along with the lack of *rmpA2*, a second copy of this gene found in the SGH10 virulence plasmid, could explain the lack of hypermucoviscosity in VA585-22. Previous reports indicated that not all the hypervirulent strains are hypermucoviscous, with deletions in *rmpA* or *rmpA2* a common feature, and thus, this trait is not a good hvKp predictor [8, 22, 23].

The second plasmid, pVA585-22_54 (~ 54 kbp, IncN) (Fig. 1, right side), was highly similar to a potentially conjugative plasmid carrying *bla*_KPC−2_ reported in a multispecies outbreak in Chile [24], which was also found with a high prevalence in other *K. pneumoniae* clinical isolates from Chile and South America [25]. However, pVA585-22_54 differed as it included a class-I integron carrying the resistance genes *bla*_VIM−1_ (VIM-1 carbapenemase), *aacA7* (aminoglycoside acetyltransferase), *dfrA1* (dihydrofolate reductase), and *aadA1* (aminoglycoside nucleotidyltransferase). This corresponds to the first report in Chile of hvKp producing two carbapenemases encoded in a single plasmid, along with other resistance determinants.

### Phylogenomic relationships with other CG23 hvKp and carbapenemase plasmids mediating convergence

**Fig. 2:**
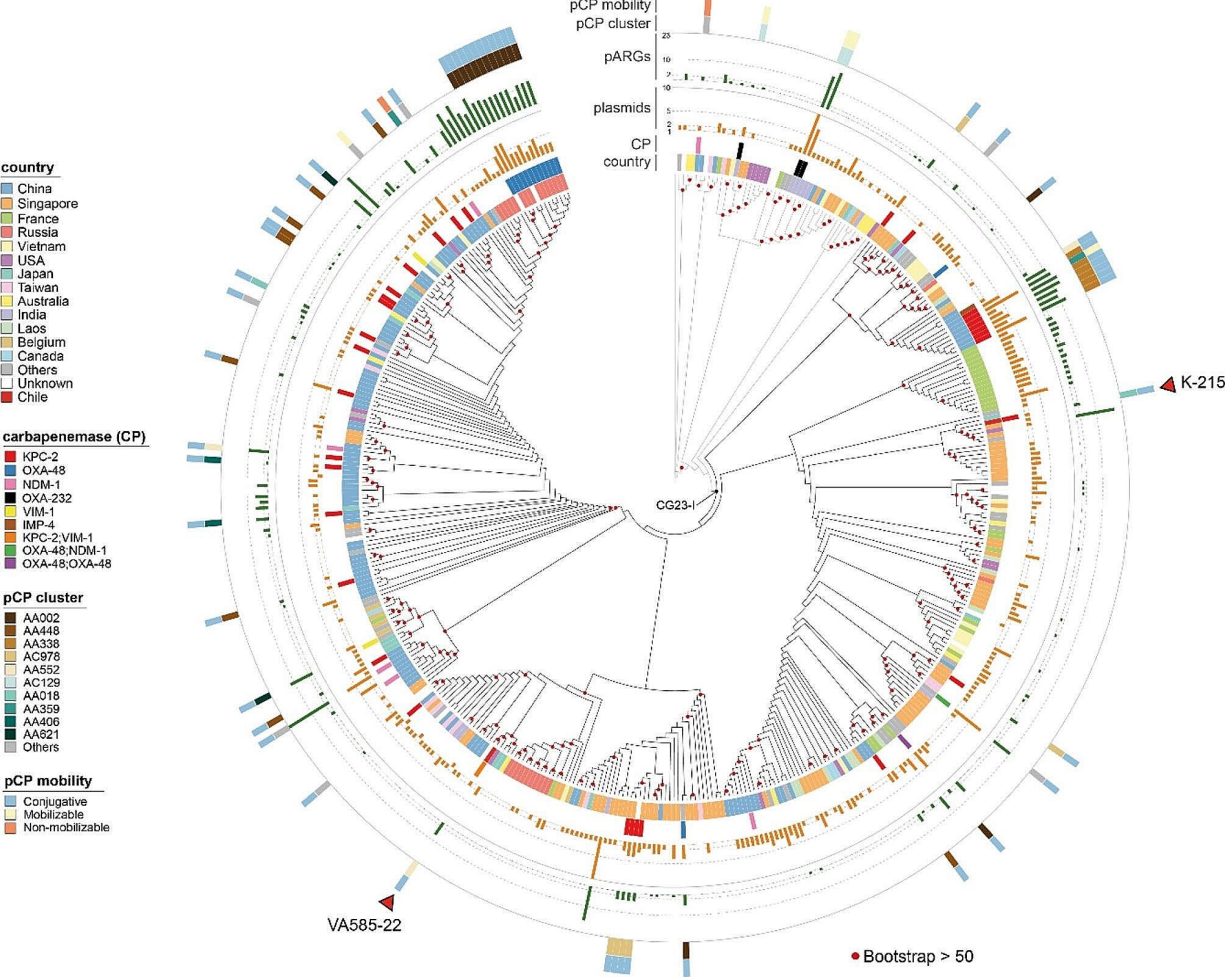
Phylogenomic relationships, antimicrobial resistance, and carbapenemase-encoding plasmids among VA585-22 and other CG23 hvKp. Phylogenetic tree inferred from the core genome multiple sequence alignment (cg-MSA) using 629 loci from the scgMLSTv2 scheme. The distances were ignored for more clarity. The tracks show (inner to outer) the country of origin, the presence/class of carbapenemases (CP), the number of plasmids identified in each isolate, the number of plasmid-encoded antibiotic resistance genes (pARGs), the carbapenemases plasmid (pCP) cluster (as defined by the MOB_typer tool), and its predicted mobility. The two carbapenem-resistant ST23 *K. pneumoniae* isolated in Chile are indicated with a red triangle

We investigated the phylogenetic relationships of VA585-22 with other 434 genomes of CG23 *K. pneumoniae* (most of the available from the NCBI database) isolated from more than 35 countries (Table S2), including the KPC-2-producing hvKp strain K-2157 isolated recently in Chile [26]. Although a few other reports described CR-hvKp from South America [17, 18, 27], no genome sequences of these isolates were published. Classical seven-gene and core-genome multilocus sequence analysis (using the 629-loci scgMLSTv2 scheme [28]) indicated that VA585-22 belongs to ST23, specifically to the globally disseminated CG23-I, clustering with isolates from diverse geographical origins, mainly from the USA, Australia, Japan, China, Taiwan, and Singapore (Fig. 2). The K-2157 strain clustered in a separate branch from VA585-22 (diverging by 17 allelic mismatches), suggesting it would correspond to a different clone. For clarity, distances in the tree shown in Fig. 2 are omitted. A tree including the distances and the accession numbers of the genomes is shown in Fig. S3.

Given the relevance of carbapenem resistance in hvKp, we compared the carbapenemase-coding genes and plasmids found in VA585-22 and our set of 435 CG23 isolates. Fifty-eight strains (13.3%) encoded one carbapenemase, while three strains (0.69%) encoded two (including VA585-22, the only producing KPC-2 and VIM-1) (Fig. 2). In 58 of these 61 isolates (95%) the carbapenemase was encoded in a plasmid, and isolates with more plasmids tended to have more acquired ARGs, highlighting the relevance of these mobile elements in hvKp multi-drug resistance.

Using the MOB-Suite tools, we typed and clusterized the plasmids encoding carbapenemases. The most frequent carbapenemase was KPC-2, carried by several plasmid clusters (11) and CG23-I subclades from different countries, leading the cluster AA448 (IncU), also carrying the mercury resistance genes *mer*, mainly from Chinese isolates (Fig. 2, Fig. S4). On the other hand, VA585-22 and a 2017 Chinese isolate (NZ_CP096241) were the only CG23 strains carrying an AA552 (IncN) plasmid encoding KPC-2. Conversely, the K-2157 KPC-2 plasmid (cluster AA018; ~114-kbp; IncFIB, IncFII), also including *bla*_TEM−1_, *bla*_OXA−9_, and the *mer* operon, was highly similar to pDHQP17016, previously found in an ST23 isolate from the USA [16, 18]. We found a similar plasmid in a 2018 CG23 isolate from Poland (GCF_022748855). Other relevant KPC-2 plasmids identified in CG23 hvKp included the cluster AC978, which is highly stable and transmissible and has become dominant among Enterobacterales in Singapore [29].

Other carbapenemases showed a narrower distribution among CG23 hvKp. VIM-1 was found only in three isolates, in VA585-22, in one from China, and one from Poland, the two latter bearing AA621 plasmids (IncA). OXA-48 was found only in AA002 plasmids (IncL/M), mainly from Russian isolates, OXA-232 was found exclusively in AC129 plasmids (rep_cluster_1195) from India, while NDM-1 was found in four plasmid clusters, whileIMP-4 was found in only one isolate bearing an AA552 plasmid. Most of the carbapenemase plasmids described above were predicted as conjugative.

### Dissemination of the KPC-2 VIM-1 plasmid VA585_22–54 by conjugation

We tested the possible conjugative dissemination of pVA585-22_54, setting up a conjugation assay using an *E. coli K12* Gm^R^ strain as the recipient. Eighteen transconjugants were obtained, and two were selected for characterization (K12-1 and K12-2). Remarkably, the acquisition of this single plasmid conferred to the *E. coli* recipients resistance to all the beta-lactams tested, pointing out the broad spectrum of beta-lactam antibiotics targeted by this carbapenemases combination. Accordingly, PCR amplification confirmed the acquisition of *bla*_KPC−2_ and *bla*_VIM−1_ genes by K12-1 and K12-2, while their expression was confirmed by immunochromatography (Table 1). Thus, contrary to that observed with the other hvKp strain isolated in Chile (K-2157) [26], VA585-22 could conjugate the plasmid pVA585-22_54 to *E. coli*, conferring dual carbapenemase production and multi-drug resistance.

## Conclusions

**Fig. 3:**
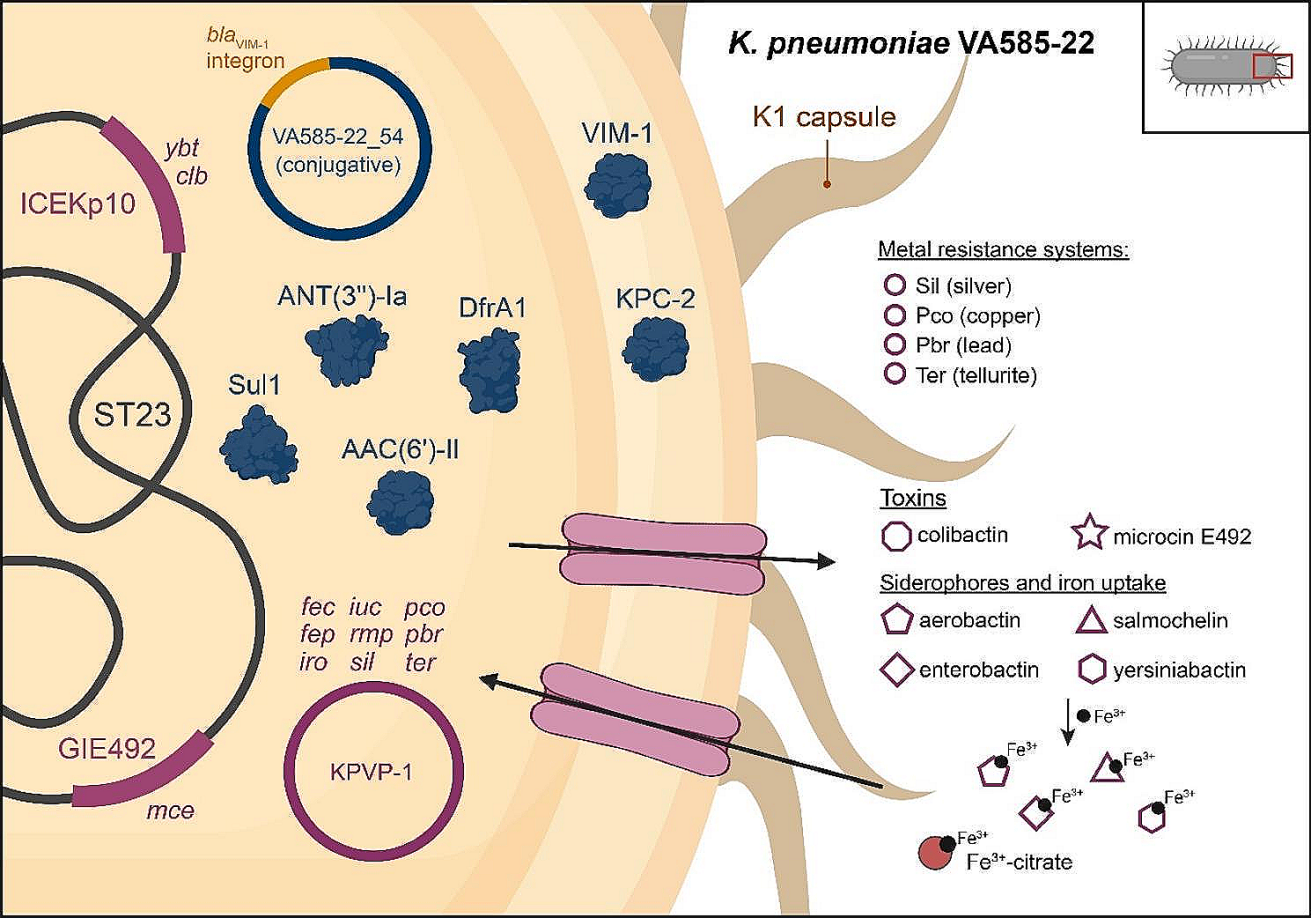
Schematic representation of the main resistance genes, virulence factors, and mobile genetic elements in the CR-hvKp strain *K. pneumoniae* VA585-22. The virulence and metal resistance factors encoded in GIE492 (*mce*, MccE492 and salmochelin), ICEKp10 (*ybt*, yersiniabactin; *clb*, colibactin), and pKPVP-1 (*iuc*, aerobactin; *iro*, salmochelin; *fep*, iron transporter; *fec*, Fur-dependent system for iron uptake) are colored in magenta. For simplicity, only one export and one import protein complexes are depicted. The virulence plasmid also includes the *sil*, *pco*, *pbr*, and *ter* genes linked with resistance to copper, silver, lead, and tellurite, respectively, and the *rmp* genes linked to capsule upregulation. The antibiotic resistance proteins encoded in pVA585-22_54 are presented in dark blue (VIM-1 and KPC-2 carbapenemases, DfrA1 trimethoprim-resistant dihydrofolate reductase, ANT(3’’)-Ia aminoglycoside nucleotidyltransferase, AAC(6’)-II aminoglycoside acetyltransferase, and Sul1 sulfonamide-resistant dihydropteroate synthase). Created with BioRender.com

We characterized phenotypically and at the genomic level the CG23-I (ST23) hvKp strain VA585-22 isolated in Chile (main features summarized in Fig. 3), corresponding to the first report of hvKp co-producing KPC-2 and VIM-1 carbapenemases encoded in a single conjugative plasmid. This plasmid likely arose from incorporating a class-I integron carrying *bla*_VIM−1_ and other resistance genes into an IncN KPC-2 plasmid highly disseminated in South America and previously found in Chile. Considering its high transmissibility and that it confers resistance to most beta-lactams, this double carbapenemases plasmid, and especially convergent hvKp comprising it, are of utmost concern.

## Methods

### Bacterial strains and culture conditions

*K. pneumoniae* VA585-22 was collected by the Chilean Public Health Institute. *K. pneumoniae* SGH10 was kindly provided by Prof. Yunn Hwen Gan (National University of Singapore). The SGH10 D*wcaJ* mutant derivative was constructed following the scarless site-directed mutagenesis procedure described previously [21]. The *K. pneumoniae* strains were grown overnight at 37 °C in LB, Mueller-Hinton, or blood agar plates, supplemented with meropenem (4 µg/mL) when required. The *E. coli* K12 Gm^R^ recipient strain (spontaneous mutant from A. Marcoleta’s laboratory collection) for conjugation experiments was grown at 37 °C in LB or Mueller-Hinton agar plates supplemented with gentamicin (10 µg/mL).

### Mucoviscosity assessment

Mucoviscosity was evaluated using the string test and a low-speed sedimentation assay, as described previously [21]. For both assays, bacterial isolates were grown overnight in blood agar at 37 °C. For sedimentation, an appropriate amount of biomass was suspended in sterile PBS and then diluted to obtain 5 mL of bacterial suspensions adjusted to an OD_600 nm_ = 4, poured into sterile 15 mL conical tubes. Then, the tubes were centrifuged for 5 min at 1000 xg, measuring the OD_600 nm_ of the supernatant (diluting when required). A higher OD_600 nm_ (close to the value before centrifuging) indicated hypermucoviscosity, as these strains tend to float and remain in the supernatant. For the string test, isolated colonies from VA585-22, or the control strains SGH10 and SGH10 D*wcaJ*, were stretched with a bacteriology inoculation loop. The test was positive if a viscous string with a length > 5 mm is obtained [30].

### Bacterial conjugation

A standard conjugation assay in *E. coli* was carried out as previously described [29]. Briefly, the donor VA585-22 and recipient *E. coli* K12-Gm^R^ strains were grown overnight in LB broth supplemented with meropenem and gentamicin, respectively. Conjugation was performed in a 0.22 μm sterile cellulose ester membrane filter (Merck, Germany) deposited over an LB agar plate, pouring a 1:1 ratio (50 µL) of donor and recipient strains. The plate with the filter was incubated for 3 h at 37ºC, and then half of the membrane was mixed with 3 mL of LB broth and vortexed briefly. Then, 100 µL of this suspension were plated onto LB-Gm-Mem and incubated at 37 °C overnight to select transconjugants (*E. coli* K12- Gm^R^-Mem^R^).

### Detection of carbapenemase genes and carbapenemase production

Transconjugants were analyzed by PCR using Platinum™ Taq DNA polymerase (Invitrogen, ThermoFischer Scientific, USA) and specific primers for *bla*_*KPC−2*_ and *bla*_*VIM−1*_ (Table S3), as previously described [31]. Carbapenemase expression was confirmed by immunochromatography using NG-Test Carba 5 assay, according to the manufacturer’s instructions [32].

### Antimicrobial susceptibility test

The Kp VA585-22 isolate, *E. coli* K12-Gm^R^ and two *E. coli* K12 transconjugant clones (Gm^R^-Mem^R^) were studied by epsilometry (E-test, Biomerieux) and broth microdilution using BD Phoenix™ System (Becton Dickinson, USA), according to M100 Performance Standards for Antimicrobial Susceptibility Testing, 33rd edition [33].

### Genome sequencing, assembly and annotation

Genomic DNA was extracted using the GeneJET Kit (Thermo Scientific) and quantified using a Qubit fluorometer (Invitrogen). Illumina sequencing (100-bp paired-end) was done with the TruSeq Nano DNA kit and a Hiseq4000 machine (hired to Seqcenter, Inc., USA). Nanopore sequencing was done using the Rapid Barcoding kit (SQK-RBK004) and a FLO-MIN106 flow cell in a MinION device.

Illumina reads were trimmed and quality filtered using fastp v0.23.2 [34]. Nanopore sequencing data was base-called with Guppy v6.5.7 + ca6d6af with the dna_r9.4.1_450bps_sup.cfg model, and the ONT reads were then quality-filtered using Filtlong v0.2.1 (https://github.com/rrwick/Filtlong), and subsampled into 12 read sets using Trycycler v0.5.4 [35]. Four read sets were assembled using Flye v2.9.2-b1786 [36], four using raven v1.8.1 [37], and four using minimap2 v2.24-r1122 [38], miniasm v0.3-r179 [39] and minipolish v0.1.2 [40]. All 12 assemblies were used as input to generate a consensus long-read assembly following the trycycler v0.5.4 pipeline. The long-read consensus assembly was polished using Medaka v1.7.2 (https://github.com/nanoporetech/medaka) with the r941_min_sup_g507 model. The Medaka-polished assembly was then polished using the filtered Illumina reads with Polypolish v0.5.0 [41] and POLCA (MaSuRCA v4.0.5) [42]. The assembly was evaluated using QUAST v5.0.2 [43] and CheckM [44], and annotated with Bakta v1.8.1 [45]. Gene organization analysis was performed with Clinker [46]. Nucleotide sequence alignment and visualization was performed with Clustal Omega [47] and Jalview [48].

### CG23 *K. pneumoniae* database construction

17,612 *K. pneumoniae* species complex (KpSC) genomes were downloaded from the NCBI RefSeq database on April 5th, 2023. Upon filtering out genomes of poor quality, with ambiguous nucleotide bases, and non-standard *Klebsiella* genomes (≥ 1,000 contigs, genomic size ≤ 4.5 Mbp or ≥ 6.5 Mbp, > 59% GC content, < 96% Average Nucleotide Identity to reference KpSC genomes), 11,817 genomes were selected for further analysis. Additionally, we included *K. pneumoniae* VA585-22, 119 genomes from the Antibiotics for *Klebsiella* Liver Abscess (A-KLASS) cohort (PRJNA956314) [49], 365 *K. pneumoniae* isolates from a bloodstream infection cohort (BSI) from Asia [50], 34 *Klebsiella* genomes from the Murray Collection in the pre-antibiotic era [51], and 97 CG23 genomes analyzed by Lam et al. (2018) [7]. The resulting 12,433 *K. pneumoniae sensu stricto* species genome set was subjected to multilocus sequence typing (MLST) and screened for relevant information using Kleborate v2.3.2 [52]. The 629-loci cgMLST scheme, scgMLSTv2 [28], was used to search for alleles in all 12,433 Kp1 genomes using the BLASTn v2.13.0 algorithm [53] under a 95% identity and 95% coverage threshold. The best hit for each allele was selected according to the local alignment reported bitscore. Selected alleles were used to generate cgMLST profiles for each genome. The cgMLST profiles of the 12,433 Kp1 genomes and 34,055 reference profiles were used as input for the LINcoding algorithm [28] to assign cgLIN codes to each genome. 435 genomes belonging to CG23 according to the cgLIN code were kept for further analysis.

### Phylogenomic analysis

A core genome multiple sequence alignment (cg-MSA) was constructed using 629 loci previously defined [28] for the 435 CG23 genomes. The individual gene sequences were aligned in global pair mode using MAFFT v7.471 [54]. A phylogenetic tree was inferred using IQ-TREE v2.2.2.3 [55] with 139,808 set as seed and 1,000 non-parametric bootstraps. The best nucleotide substitution model, GTR + F + I, was predicted using ModelFinderPlus [56].

### Plasmid typing, clustering, and prediction of mobile genetic elements

The 435 CG23 genomes were used as input for the MOB-suite v3.1.4 [57] mob_recon module to reconstruct and type plasmids with default parameters. All plasmid sequences were used as input for NCBI AMRFinderPlus v3.11.14 [58] to identify antimicrobial, metal, and biocide resistance genes (Database version: 2023-08-08.2). Insertion sequences were predicted in reconstructed plasmids using ISEScan v1.7.2.3 [59]. Complete integrons, CALINs and integron integrases were predicted using IntegronFinder v2.0.2 [60].

### Electronic supplementary material

Below is the link to the electronic supplementary material.

**Supplementary Material 1:** **Table S1.** VA585-22 genome sequence stats. **Table S3.** Primers used for carbapenemase genes detection. **Figure S1.** Hypermucoviscosity evaluation through low-speed sedimentation and the string test. **Figure S2.** Gene organization and sequence conservation of the rmpADC locus and its surroundings. **Figure S3.** Phylogenetic relationships among VA585-22 and other 434 K. pneumoniae CG23 genomes. **Figure S4.** Plasmids carrying carbapenemase genes present in CG23 hvKp genomes

**Supplementary Material 2:** **Table S2.** Accession numbers and relevant information of the CG23 *K. pneumoniae* isolates included in the phylogenomic analysis

## Data Availability

The complete genome assembly of *K. pneumoniae* VA585-22 was deposited in the NCBI genome database under the Bioproject number PRJNA1020101 and the assembly accession GCF_032253935.1.
