# Supplementary material for: Carbapenem-resistant hypervirulent ST23 Klebsiella pneumoniae with a highly transmissible dual-carbapenemase plasmid in Chile
Source: Biol Res. 2024 Mar 12;57:7. doi: 10.1186/s40659-024-00485-2 (PMC10929235; doi:10.1186/s40659-024-00485-2)
Supplement: Supplementary file 1 — Supplementary Material 1: Table S1. VA585-22 genome sequence stats. Table S3. Primers used for carbapenemase genes detection. Figure S1. Hypermucoviscosity evaluation through low-speed sedimentation and the string test. Figure S2. Gene organization and sequence conservation of the rmpADC locus and its surroundings. Figure S3. Phylogenetic relationships among VA585-22 and other 434 K. pneumoniae CG23 genomes. Figure S4. Plasmids carrying carbapenemase genes present in CG23 hvKp genomes [file 40659_2024_485_MOESM1_ESM.pdf]

Carbapenem-resistant hypervirulent ST23 *Klebsiella pneumoniae* with a highly transmissible dual-carbapenemase plasmid in Chile

Gálvez-Silva M[1,+], Arros P[1,+], Berríos-Pastén C[1], Villamil A[3], Rodas PI[3], Araya I[3], Iglesias R[3], Araya P[3], Hormazábal JC[3], Chen Y[4], Gan Y-H[4], Chávez FP[2], Lagos R[1], Marcoleta AE[1,*].

[1]Grupo de Microbiología Integrativa, Laboratorio de Biología Estructural y Molecular BEM, Departamento de Biología, Facultad de Ciencias, Universidad de Chile. Las Palmeras 3425, Ñuñoa, Santiago, Chile.
[2]Laboratorio de Microbiología de Sistemas, Departamento de Biología, Facultad de Ciencias, Universidad de Chile. Las Palmeras 3425, Ñuñoa, Santiago, Chile.
[3]Instituto de Salud Pública. Marathon 1000, Ñuñoa, Santiago, Chile.
[4]Yong Loo Lin School of Medicine, National University of Singapore, MD7, 8 Medical Drive, Singapore.

SUPPLEMENTARY MATERIAL

Table S1. VA585-22 genome sequence stats.

Feature	Value
Amount of sequenced bases (Illumina)	317,287,242 bp
Amount of sequenced bases (Nanopore)	438,611,421 bp
Total assembly size	5,615,879 bp
Average coverage depth	137.72X
GC content	57.11%
Number of contigs	3 circular
CheckM completeness	100%
CheckM contamination	1.18%
NCBI accession number	(submission in process)
Antibiotic resistance genes	bla_{KPC-2}, bla_{VIM-1}, bla_{TEM-1}, *aacA7*, *dfrA1*, *aadA1*, *smr*, *qacEdelta1*, *sul1*
Metal resistance genes	*silERSCBAP*, *pcoABCDRSE*, *pbrRABC*,
Virulence genes	*iucABCDiutA*, *fepBCD*, *rmpADC*, *iroBCDN*, *fecIRA*, *feoB, ybt* genes*, clb* genes

Table S2. Accession numbers and relevant information of the CG23 K. pneumoniae isolates included in the phylogenomic analysis (provided as a separate spreadsheet).

Table S3. Primers used for carbapenemase genes detection.

Gene		Sequence	Size (bp)	Annealing Temperature (ºC)
*bla*KPC	F	5′-AACAAGGAATATCGTTGATG-3′	916	55º
	R	5′-AGATGATTTTCAGAGCCTTA-3′		
*bla*VIM	F	5′-GGTCTAYWTGACCGCGTCTR-3′	700	55º
	R	5′-STGCTTCCGGGTAGTGTTK-3′		

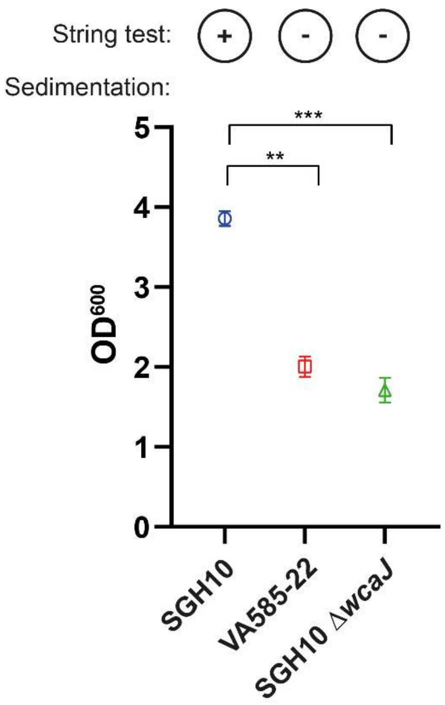

Figure S1. Hypermucoviscosity evaluation through low-speed sedimentation and the string test. For the sedimentation assay, bacterial suspensions of VA585-22, the model hvKp strain SGH10 (hypermucoviscous), or the capsule-null mutant SGH10 Δ*wcaJ* (non-hypermucoviscous) were prepared at a final optical density at 600 nm (OD^{600}) = 4, and then centrifuged for 5 min at 1000xg. The OD^{600} of the supernatant was then measured and plotted. The symbols and error bars correspond to the average and standard deviation from three independent experiments. **P >0.001, ***P >0.0001. For the string test, each strain was grown overnight in blood agar.

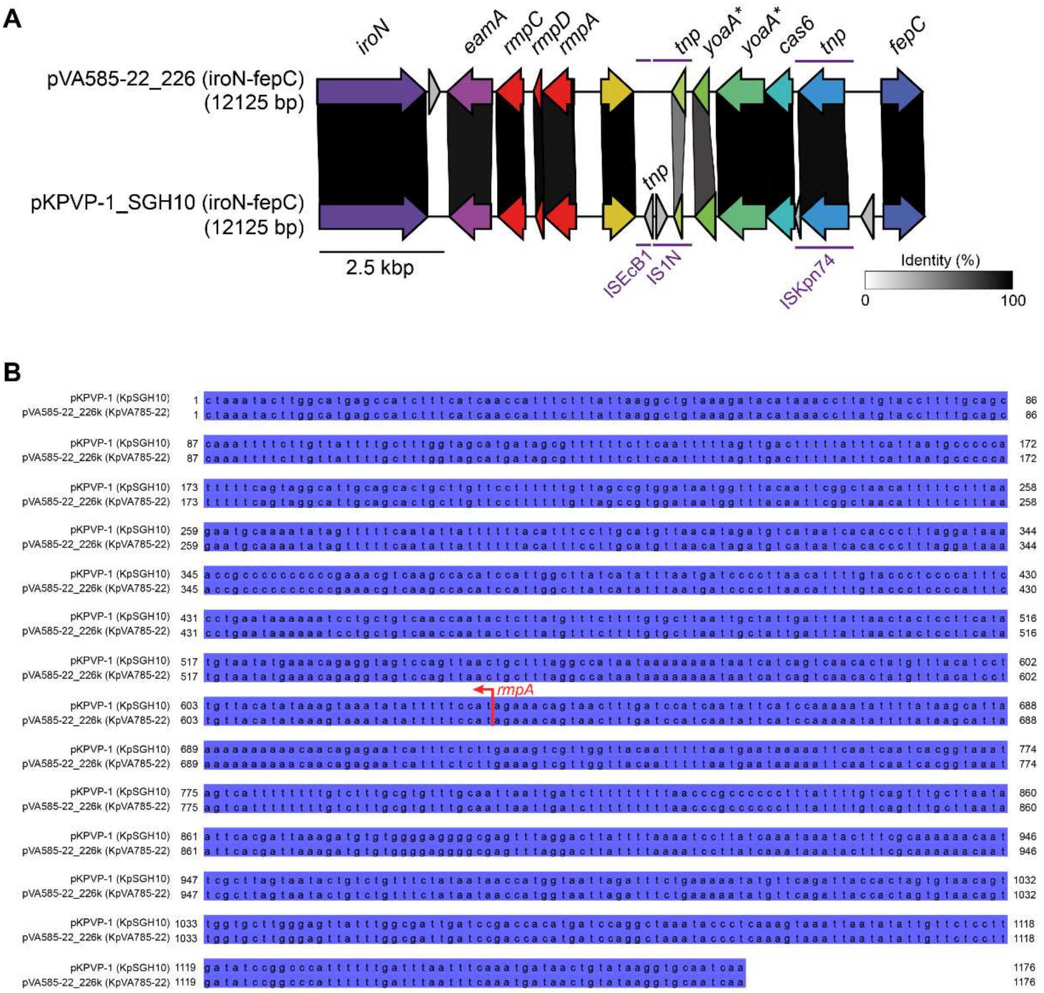

Figure S2. Gene organization and sequence conservation of the *rmpADC* locus and its surroundings. A. *rmpADC* gene organization and context in pVA585-22_226 (*K. pneumoniae* VA585-22) and pKPVP-1 (*K. pneumoniae* SGH10). B. Nucleotide sequence alignment fo the region comprising *rmpA* and its upstream intergenic region (including the *rmp* operon promoter). The nucleotide bases are colored according to percentage identity.

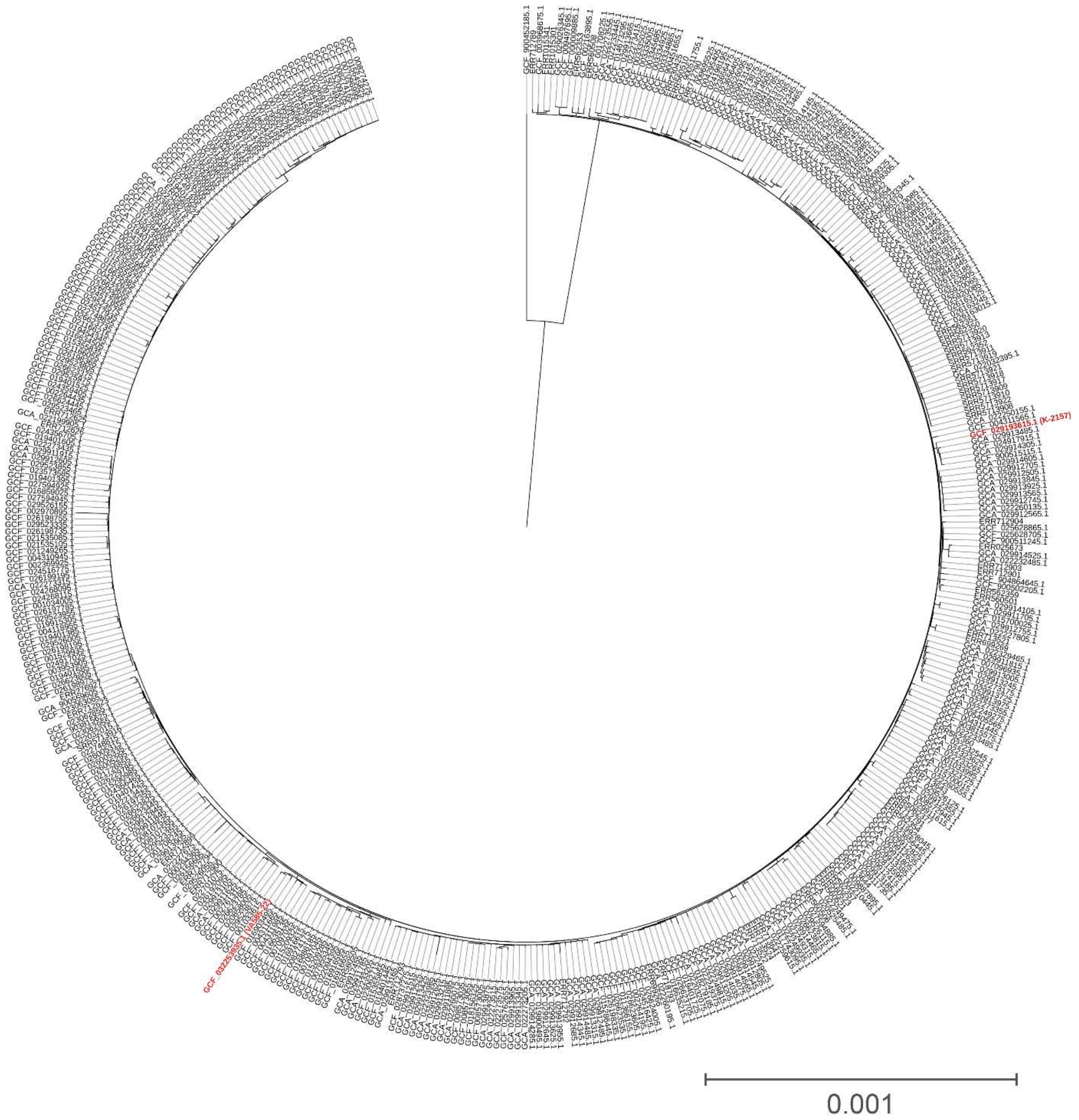

Figure S3. Phylogenetic relationships among VA585-22 and other 434 *K. pneumoniae* CG23 genomes. Maximum likelihood distance tree inferred from the multiple sequence alignment of the 629 loci defined for the scgMLSTv2 scheme. The two CR-hvKp isolated in Chile are colored in red.

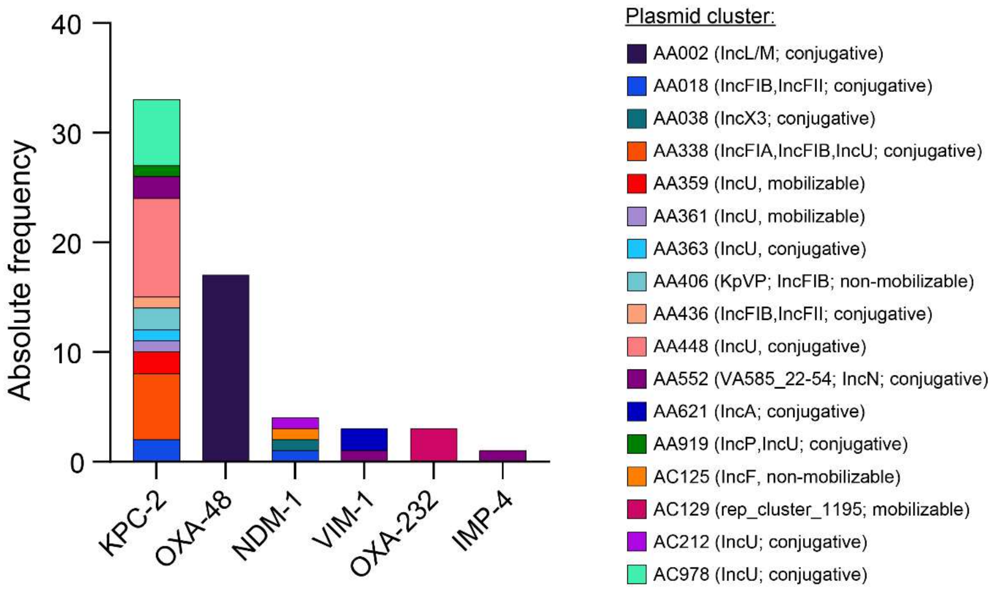

Figure S4. Plasmids carrying carbapenemase genes present in CG23 hvKp genomes. VA585-22 and other 434 *K. pneumoniae* CG23 genomes were screened for plasmids encoding carbapenemases, which were then typed and clusterized using the MOB-suite tools. The barplot shows the number of plasmids from each cluster carrying the respective carbapenemase gene.
